# Does the Disease of the Person Receiving Care Affect the Emotional State of Non-professional Caregivers?

**DOI:** 10.3389/fpsyg.2019.01144

**Published:** 2019-05-15

**Authors:** Patricia Otero, Ángela J. Torres, Fernando L. Vázquez, Vanessa Blanco, María J. Ferraces, Olga Díaz

**Affiliations:** ^1^Department of Psychology, University of A Coruña, A Coruña, Spain; ^2^Department of Psychiatry, Radiology, Public Health, Nursing and Medicine, University of Santiago de Compostela, Santiago de Compostela, Spain; ^3^Department of Clinical Psychology and Psychobiology, University of Santiago de Compostela, Santiago de Compostela, Spain; ^4^Department of Evolutive and Educational Psychology, University of Santiago de Compostela, Santiago de Compostela, Spain; ^5^Department of Social, Basic and Methodological Psychology, University of Santiago de Compostela, Santiago de Compostela, Spain

**Keywords:** emotional state, mental health, caregiver, disease, diagnosis, care-recipient

## Abstract

Research on mental health of non-professional caregivers has focused on caregivers of people with specific diseases, especially dementia. Less is known about caregivers of people with other diseases. The aims of this study were (a) to determine the caregivers’ emotional state in a random sample of caregivers of people in situations of dependency, (b) to analyze the association between each disease of the care-recipient (a variety of 23 diseases included in the International Classification of Diseases) and the emotional state of the caregiver, and (c) based on the theoretical model, to analyze the relationship of the different study variables in the appearance of the emotional distress of the caregiver. A sample of 491 non-professional caregivers was selected randomly (89.0% women, average age 55.3 years). Trained psychologists collected sociodemographic and care-related characteristics and evaluated the global emotional distress, somatic symptoms, anxiety-insomnia, social dysfunction, depression, probable mental disorder case, self-esteem, and social support. It was found that (a) the caregivers showed moderate emotional distress, and 33.8% presented a probable mental disorder. (b) Caring for a care-recipient with cat’s cry syndrome or epilepsy was related to suffering from social dysfunction, and caring for a care-recipient with autism was related to having a probable mental health case. (c) Social support mediated the relationship between social class, daily hours of care, monthly family income, self-esteem and global emotional distress. There is an important impact on the emotional state of the caregivers. This impact was similar in caregivers of care-recipients with different diseases, except in caregivers caring for a care-recipient with cat’s cry syndrome or epilepsy (related to social dysfunction), and in caregivers caring for a care-recipient with autism (related to having a probable mental health case).

## Introduction

It is estimated that around 350 million people in the world are in situations of dependency (Harwood et al., 2004). Approximately 301 different diagnoses have been identified in which assistance in daily life activities are required (Global Burden of Disease Study 2013 Collaborators, 2015), and this assistance is generally provided by a relative (Solé-Auró and Crimmins, 2014). However, scientific literature has clearly documented the negative consequences that care has on non-professional caregivers’ mental health (Vázquez et al., 2018). Depression, anxiety, insomnia, and social dysfunction are the most common psychological problems (Clark and Bond, 2000; Torres et al., 2015; Liu et al., 2017).

Until now, this research has focused on the effects of those who take care for family members suffering from dementia, and numerous psychological interventions have been specifically developed for them (Sörensen and Conwell, 2011). One reason for this is that care-recipients with dementia are a large population, around 50 million worldwide (World Health Organization [WHO], 2017). Furthermore, dementia caregivers may be more at risk of suffering adverse mental health effects due to people with dementia exhibit severe disruptive behaviors, cognitive impairment, mood changes, require more supervision, express less gratitude, and may exhibit aggressiveness (Müller-Spahn, 2003). To a lesser extent, psychological effects of caring for people with other specific diseases, such as cancer or stroke have been studied (e.g., Han and Haley, 1999; Romito et al., 2013).

In addition, the effect of particular diseases on the caregiver’s psychological well-being has yet to be fully explored. According to the cognitive theory of stress (Lazarus and Folkman, 1984), later adapted by Pearlin et al. (1990) specifically for caregivers, emotional distress in caregivers is a consequence of a process comprising a number of interrelated conditions, including sociodemographic characteristics, primary and secondary stressors to which they are exposed and the resources of caregivers. Primary stressors include problems related directly to caregiving, such as the clinical manifestations of the disease of the person receiving care (e.g., alterations of memory and behavior changes in dementia) and others like degree of autonomy of the care-recipient, duration of care or daily hours of care. The primary stressors can be assessed as threatening by caregivers and create conditions under which emotional distress may occur. Secondary stressors include stress experienced in roles outside of caregiving (e.g., employment status, income) and psychological stressor (e.g., loss of self-esteem). The resources of caregivers involve their perceived social support. Thus, the extent to which caregivers experience distress depends not only on primary stressors, such as the type of disease of the care-recipient, but also on their appraisal style and resources in managing stressors.

Available empirical data about differences in emotional state between caregivers of people with different diseases are limited and inconclusive. On the one hand, Clipp and George (1993) compared 272 caregivers of care-recipients with dementia and 30 of care-recipients with cancer. They found that dementia caregivers had significantly higher stress, burden and negative affect compared to caregivers of people with cancer. Similarly, Ory et al. (1999) compared 320 dementia caregivers with 1,178 caregivers of people without dementia and found that caring for a person with dementia has more adverse effects in terms of physical and emotional strain, physical and mental problems, free time and family conflict.

On the other hand, Crespo et al. (2005), when comparing caregivers of 66 demented persons and 42 care-recipients without cognitive impairment, found no evidence that dementia caregivers had more depressive and anxiety symptoms than those who care for care-recipients without dementia. Additionally, Papastavrou et al. (2012) in a sample of 415 caregivers (172 of people with dementia, 113 with schizophrenia and 130 with cancer) reported that caregivers of people with cancer were more depressed compared to caregivers of schizophrenia and dementia care-recipients, while dementia caregivers reported the highest levels of burden. Finally, in 202 caregivers (85 of people with dementia, 44 with physical diseases, 28 with neurological diseases, 24 with mental disorder and 21 with stroke), Loi et al. (2015) found higher levels of depressive symptomatology and burden in caregivers of people with physical diseases compared to dementia caregivers.

Furthermore, these studies have methodological limitations that hinder the generalization of results. They compared specific diseases (Clipp and George, 1993; Ory et al., 1999; Crespo et al., 2005; Papastavrou et al., 2012) excluding many diseases in the International Classification of Diseases and Related Health Problems (ICD-10; World Health Organization [WHO], 2015) that are known to generate dependence; used convenience samples (Clipp and George, 1993; Papastavrou et al., 2012; Loi et al., 2015), used comparison groups with a small sample size (Clipp and George, 1993; Crespo et al., 2005; Loi et al., 2015) and did not use standardized instruments (Ory et al., 1999). The aims of our study were (a) to determine the caregivers’ emotional state in a random sample of caregivers of people in situations of dependency, (b) to analyze the association between each care-recipient disease (from a variety of diseases included in the ICD-10) and the caregiver emotional state, and (c) based on the theoretical model by Pearlin et al. (1990), to analyze the relationship of the process stress variables (sociodemographic variables, primary and secondary stressors, caregiver resources) in the appearance of the emotional distress of the caregiver. We expected a moderate emotional distress in the caregivers’ sample. We also expected that the disease of the care-recipient is not associated to the caregiver’s emotional state. Lastly, it is expected that in the caregiver stress process generated by the care situation, sociodemographic characteristics (i.e., age, gender, marital status, educational level and social class of the caregiver, relationship with the care-recipient, and gender of the care-recipient) would be related to primary stressors (i.e., the care-recipient’s disease, degree of autonomy of the patient, duration of care and daily hours of care), and that primary and secondary stressors (i.e., employment, monthly family income, caregivers’ self-esteem) would be related to caregivers’ emotional state. In addition, we expected that all of these relationships would be mediated by the available resources (i.e., social support).

## Materials and Methods

### Participants

A cross-sectional study design was conducted. Participants were selected from January 2015 to January 2016 from the official register of the Ministry of Labor and Welfare of Galicia, a region of 29,434 km^2^ in northwest Spain with 2,732,347 inhabitants. This register was created on the basis of the Spanish Law 39/2006 of December 14, the Promotion of Personal Autonomy and Care for People in Situations of Dependency Act (Ley 39/2006, 2006). The law is designed to regulate the basic conditions of equality and care for people in situations of dependency. It is the official register of non-professional caregivers of the region, includes caregivers of dependent people with different diseases included in the ICD-10, and contains contact details of caregivers. For the purpose of this study and through a previous agreement with the Ministry of Labor and Welfare of the Galician Regional Government, this register was used to extract the sample and contact details to contact caregivers by mail or telephone. In order to reduce selection bias, a sample of 20 localities in the Region of Galicia was randomly selected stratified by area type [rural (<2000 inhabitants) or urban (≥2000 inhabitants)] and province (Coruña, Lugo, Pontevedra or Orense). The sample was then randomly selected by simple random sampling by an independent statistician.

To participate in this study, participants had to: (a) be a non-professional caregiver of a person with recognized dependency by the official administration; (b) live with the care-recipient; (c) be 18 years of age or older; and (d) provide informed consent. We excluded participants who: (a) presented any communication difficulty (e.g., not knowing how to read or write) or any condition that made evaluation impossible (e.g., significant cognitive or visual impairment); (b) took care of a care-recipient with a terminal prognosis within the next 6 months; (c) had received psychological or pharmacological treatment in the last 2 months.

**FIGURE 1:**
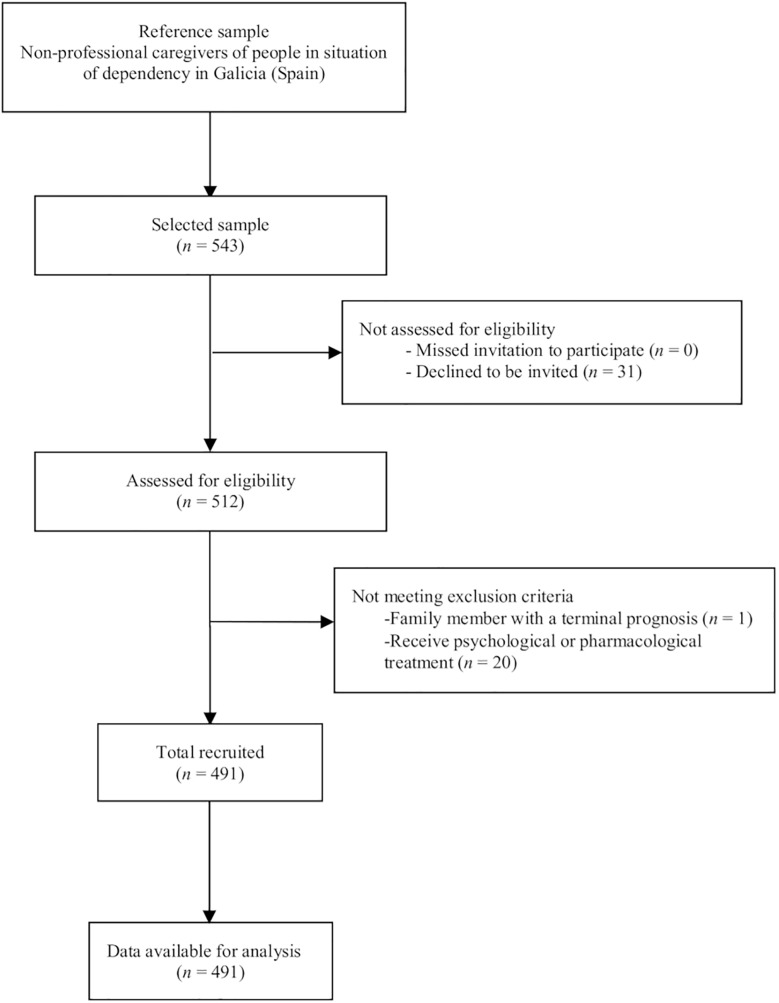
STROBE (Strengthening the Reporting of Observational Studies in Epidemiology) flow diagram.

In the total of 543 caregivers invited to participate, the response rate was 94.3%. Thirty-one caregivers (5.7%) declined to participate, and 21 (4.1%) were excluded because the care-recipient had a terminal condition or the caregiver was receiving psychological or pharmacological treatment. To minimize participant dropout, data collection strategies for cross-sectional studies were followed (Hulley et al., 2013). The final sample was composed of 491 participants (Figure 1).

Participation was voluntary and resulted in no monetary or other compensation. The study was conducted in accordance with the Declaration of Helsinki. All participants gave written informed consent. The protocol was approved by the bioethics committee of the University of Santiago de Compostela (Spain). Any caregiver who was noted as having a probable mental disorder case was referred for further assessment and treatment to the mental health community services.

### Measures

Sociodemographic and care-related variables were collected using an questionnaire specifically developed for this study. The care-recipients’ diagnoses were based on the ICD-10 (World Health Organization [WHO], 2015) and were extracted from the official register of the Ministry of Labor and Welfare of Galicia, which were established by teams of state medical and psychological professionals after conducting complete individual assessments and considering the medical history of the care-recipient. The registered diseases included blindness, stroke, heart disease, chronic obstructive pulmonary disease, kidney disease, arthritis, physical disability, cancer, spina bifida, Down syndrome, Angelman syndrome, cat’s cry syndrome, schizophrenia, bipolar disorder, autism, Rett syndrome, West syndrome, cerebral palsy, multiple sclerosis, Parkinson’s, epilepsy, Alzheimer’s and vascular dementia. Global emotional distress and its four subscales (somatic symptoms, anxiety and insomnia, social dysfunction, and depression) were assessed with the General Health Questionnaire (GHQ-28; Goldberg and Hillier, 1979; Spanish version of Galindo et al., 2017), whose internal consistency is 0.91 for the Spanish version and 0.91 in the current study. A cutting total score of 5/6 identified probable cases of mental disorder (Godoy-Izquierdo et al., 2002). The degree of autonomy of the care-recipient was assessed with the Barthel Index (BI; Mahoney and Barthel, 1965; Spanish version of González et al., 2018), whose internal consistency for the Spanish version ranges from 0.88 to 0.92; the internal consistency is 0.88 in the present study. Self-esteem was assessed with the Rosenberg Self-Esteem Scale (RSES; Rosenberg, 1965; Spanish version of Martín-Albo et al., 2007), whose internal consistency for the Spanish version is 0.85 and for this study is 0.82. Social support was evaluated with the Duke-UNC Functional Social Support Questionnaire (DUKE-UNC; Broadhead et al., 1988; Spanish version of Cuéllar-Flores and Dresch, 2012), whose internal consistency for the Spanish version is 0.90 and 0.86 for the current study.

### Procedure

A study protocol was designed according to the World Health Organization [WHO] (2018) guidelines, describing how the study was going to be conducted. This included the objectives of the study, study design, selection of participants, assessment, statistical considerations and organization of the study ensuring the safety of the participants and integrity of the data collected. Four psychologists with 4–15 years of experience in the evaluation of emotional state were trained during 30 h to conduct the evaluations by two expert clinicians (a clinical psychologist and a psychiatrist) with more than 20 years of experience in the evaluation of mental health disorders. Prior to the current study, a pilot study was conducted with 20 randomly selected caregivers to evaluate the feasibility of the study and the competence of the interviewers. The average length of the interviews was approximately 50 min. All evaluations of the pilot study were recorded and analyzed by one of the expert clinician to assess the evaluators’ performance and to provide them with feedback.

Subsequently, caregivers were personally contacted by mail and telephone and invited to participate in the study. Each participant was assessed by the trained evaluators at a location near the participant’s home provided by social community services. All evaluations were recorded and 10% of evaluations were randomly analyzed by one of the expert clinicians to ensure study protocol integrity.

### Statistical Analyses

The analyses were performed with the statistical package SPSS for Windows (version 25.0), SPSS Amos Graphics (version 21) and the free statistical software R.

#### Relationship of Each Care-Recipient Disease to Caregivers’ Emotional State

We dichotomized each of the variables related to the diseases to analyze the specific relationship of each care-recipient disease to caregiver emotional distress (1 = presence of the disease, 0 = absence of the disease).

Next, the Mann–Whitney *U* test for independent samples (continuous variables) and the chi-square or the Fisher’s exact test or Fisher–Freeman–Halton exact test for expected values less than five (categorical variables) were used to examine if there were differences in the caregivers’ and care-related characteristics, self-esteem and social support among the diseases of the care-recipient. *Post hoc* analyses were performed using adjusted standardized residuals for the chi-square tests (Beasley and Schumacker, 1995) and using pairwise nominal independence functions for the Fisher–Freeman–Halton exact test (Mangiafico, 2015).

Subsequently, we performed multiple hierarchical regression analyses to examine whether there were significant associations between caring for a relative with every specific disease and the global emotional distress and each subscale, and logistic regression analyses were conducted to examine the relation with probable cases of mental disorder. In both cases, we controlled for the relationship with the care-recipient because differences on the caregiver’s stress depending on the relationship with the care-recipient has been demonstrate in previous studies (Neal et al., 1997; Jessup et al., 2015). We also controlled for sociodemographic and care-related variables that were significant in the previous analyses (i.e., caregiver’s age in blindness, Angelman and cat’s cry syndromes; caregiver’s gender in stroke; relationship in spina bifida, Down syndrome, Angelman syndrome, cat’s cry syndrome, autism, Rett syndrome, West syndrome and Alzheimer’s; age of the care-recipient in spina bifida, Down syndrome, Angelman syndrome, cat’s cry syndrome, autism, Rett syndrome, West syndrome, Parkinson’s and Alzheimer’s; gender of the care-recipient in Down syndrome and Alzheimer’s; degree of autonomy in Alzheimer’s; duration of care in Down and Rett syndromes, Parkinson and vascular dementia).

In addition, a Bayes factor analysis using the JZS prior for non-adjusted and adjusted linear regression (quantitative dependent variables: global emotional distress, somatic symptoms, anxiety-insomnia, social dysfunction, depression) was used to determine the probability of the null hypothesis given the data to the probability of the alternative given the data. For setting the priors, we selected none-to-moderate *a priori* differences between the two levels of the categorical variables (normal prior with location = 0 and scale = 10), and a *t* distribution for the intercept for non-adjusted and adjusted logistic regression (dichotomous dependent variable: probable mental health case). The sampling algorithm used was the NUTS sampler (Hoffman and Gelman, 2014) and 10,000 runs were used to ensure convergence. Posterior-predictive checking were performed in each model for evaluating systematic departures between simulated and real data. Analyses were performed using the R library brms (Bürkner, 2017). Jeffreys (1961) cutoffs were used for the interpretation of the Bayes factor.

#### Relationship of the Process Stress Variables in the Appearance of the Emotional Distress of the Caregiver

An exploratory path analysis was performed to study the relationships among the process stress variables in the appearance of the emotional distress of the caregiver. Specifically, we analyzed the influence of the following variables on each other and on the emotional state of the caregiver: sociodemographic characteristics (age, gender, marital status, educational level and social class of the caregiver, relationship with the care-recipient, age and gender of the care-recipient), primary stressors (care-recipient disease, degree of autonomy, duration of care, daily hours of care), secondary role stressors (employment, caregiver’s monthly family income), secondary psychological stressors (self-esteem), and caregiver resources (social support). The model was based on Pearlin et al.’s (1990) theoretical model, specified as such that the sociodemographic characteristics are related to primary stressors, and that the caregiver resources mediate the relation between sociodemographic characteristics, primary stressors, secondary stressors and global emotional distress of the caregiver. Mediation was verified with the Sobel test, the B1–B1′/B1′ ratio was used to determine the percentage of variance explained by the mediator in the relationship between the predictor and the dependent variable.

The goodness of the fit between the theoretical model by Pearlin et al. (1990) and the observed data was verified by the following indices: (a) the χ^2^ statistic (Bentler and Bonett, 1980); (b) the χ^2^/ratio; (c) the goodness of fit index (GFI; Tanaka and Huba, 1985); (d) the comparative fit index (CFI; Bentler, 1990); (e) the root mean square error of approximation (RMSEA; Steiger, 1990); and (f) the standardized root mean square residual (SRMR). Because the χ^2^ statistic is very sensitive to sample size (significance can usually be achieved at *n* ≥ 200), it must be interpreted in the context of the remaining indexes. The absence of significance in χ^2^, values >0.90 in GFI and CFI, and values between 0.05 and 0.08 for RMSEA and SRMR are considered indicators of good fit (Hu and Bentler, 1999; Byrne, 2010).

**Table 1:** Sociodemographic characteristics of the caregivers, variables pertaining the care-recipient and care situation and psychological variables of the caregivers (*n* = 491).

Variables	*n*	%
**Sociodemographic characteristics of the caregiver**		
Caregivers’ mean age *M (SD)*	55.0 (10.7)	
Caregiver’s gender		
Man	54	11.0
Woman	437	89.0
Caregiver’s marital status		
Single	116	23.6
With a partner	375	76.4
Education level		
No studies/can read and write	125	25.5
Elementary	283	57.6
High school or above	83	16.9
Caregiver’s social class		
Low/medium–low	255	51.9
Medium/medium–high	236	48.1
Employment status		
Employed	82	16.7
Unemployed	409	83.3
Caregiver’s monthly family income		
Up to €999	134	27.3
From €1,000 to €1,999	290	59.1
€2,000 and above	67	13.6
**Care-related variables**		
Relationship		
Spouse/partner	46	9.4
Son/daughter	86	17.5
Father/mother	217	44.2
Other relatives	142	28.9
Average age of the care-recipient, *M (SD)*	74.7 (23.1)	
Gender of the care-recipient		
Man	134	27.3
Woman	357	72.7
Disease of the care-recipient		
Blindness	5	1.0
Stroke	44	9.0
Heart disease	14	2.9
Chronic obstructive pulmonary disease	3	0.6
Kidney disease	5	1.0
Arthritis	10	2.0
Physical disability	13	2.6
Cancer	6	1.2
Spina bifida	6	1.2
Down syndrome	32	6.5
Angelman syndrome	6	1.2
Cat’s cry syndrome	6	1.2
Schizophrenia	20	4.1
Bipolar disorder	3	0.6
Autism	7	1.4
Rett syndrome	3	0.6
West syndrome	5	1.0
Cerebral palsy	51	10.4
Multiple sclerosis	6	1.2
Parkinson’s	31	6.3
Epilepsy	12	2.4
Alzheimer’s	166	33.8
Vascular dementia	37	7.5
Barthel index, *M (SD)*	16.7 (21.7)	
Duration of care, *M (SD)*	11.5 (9.2)	
Daily hours of care, *M (SD)*	16.4 (3.6)	
**Psychological variables of the caregivers**		
Self-esteem, *M (SD)*	31.4 (4.2)	
Social support, *M (SD)*	37.5 (10.8)	

## Results

### Characteristics of the Caregivers, the Care Situation, and Psychological Resources

As can be seen in Table 1, the average age of the caregivers was 55.0 years (*SD* = 10.7). The majority were women (89.0%), had a partner (76.4%), attended elementary school (57.6%) and declared a low or medium-low social class (51.9%). The majority of the caregivers (83.3%) was unemployed, and the 59.1% has a monthly family income of €1,000–1,999.

The majority of care-recipients (44.2%) were parents of the caregivers; their mean age was 74.7 years (*SD* = 23.1) and they were predominantly women (72.7%). The most common (33.8%) disease in the care-recipients was Alzheimer’s. In addition, care-recipients presented with high dependence (mean BI = 16.7, *SD* = 21.7). Caregivers provided care for an average of 11.5 years (*SD* = 9.2) and 16.4 h a day (*SD* = 3.6).

Regarding the psychological variables of the caregivers, the average score in self-esteem was 31.4 (*SD* = 4.2) and in social support was 37.5 (*SD* = 10.8).

### Emotional State of the Caregivers

The mean global emotional distress score was 4.7 (*SD* = 5.3). For the subscales, the mean scores were 1.3 (*SD* = 1.6) for somatization, 1.9 (*SD* = 2.2) for anxiety and insomnia, 0.9 (*SD* = 1.2) for social dysfunction and 0.6 (*SD* = 1.5) for depression. Among the caregivers, 33.8% had a probable mental disorder.

### Association Between Each Care-Recipient Disease and Caregiver Emotional State

The most prevalent diseases were Alzheimer’s (33.8%), cerebral palsy (10.4%), and stroke (9.0%), while the less prevalent were chronic obstructive pulmonary disease (0.6%), bipolar disorder (0.6%) and Rett syndrome (0.6%).

Significant differences were found in the sociodemographic profile of caregivers of people with different diseases. Specifically, caregivers for people who had suffered strokes were predominantly women (*p* = 0.044) and caregivers for people with blindness were significantly older than the average age of caregivers of people who did not suffer from blindness (*M*_age_ = 69.4 vs. *M*_age_ = 54.9, *U* = 335.50, *z* = −2.79, *p* = 0.005).

Caregivers for people with Angelman syndrome and cat’s cry syndrome were significantly younger (*M*_age_ = 44.8 and *M*_age_ = 44.0 vs. *M*_age_ = 55.2, *U* = 554,500, *z* = −2.61, *p* = 0.009; *U* = 551,500, *z* = −2.62, *p* = 0.009). Care-recipients with spina bifida, Down syndrome, Angelman syndrome, cat’s cry syndrome, autism, Rett syndrome and West syndrome were significantly younger (*M*_age_ = 25.7, *M*_age_ = 25.7, *M*_age_ = 16.0, *M*_age_ = 20.3, *M*_age_ = 25.8, *M*_age_ = 27.7, *M*_age_ = 29.2, respectively) than the average for care-recipients with other diseases (*M*_age_ = 75.3, *U* = 184.00, *z* = −3.61, *p* < 0.001; *U* = 1300.00, *z* = −7.79, *p* < 0.001; *U* = 554.500, *z* = −2.61, *p* = 0.009; *U* = 551.500, *z* = −2.62, *p* = 0.009; *U* = 472.50, *z* = −3.280; *p* < 0.001; *U* = 130.50; *z* = −2.46, *p* = 0.009; *U* = 338.00; *z* = −2.78, *p* = 0.005, respectively). These care-recipients were predominantly the children of the caregivers (*p* = 0.037; *p* = 0.025; *p* = 0.003; *p* = 0.010; *p* = 0.024; *p* = 0.022; *p* = 0.022). In addition, the duration of care was significantly longer for caregivers of people with Down syndrome and Rett syndrome (*M*_years_ = 25.6 and *M*_years_ = 27.7 vs. *M*_years_ = 11.5, *U* = 2972.00, *z* = −5.64, *p* < 0.001; *U* = 65.50, *z* = −2.72, *p* = 0.006), and persons with Down syndrome were predominantly male [χ^2^(1) = 8.896, *p* = 0.003].

Care-recipients who suffered from Parkinson’s and Alzheimer’s were significantly older (*M*_age_ = 85.4 and *M*_age_ = 86.9 vs. *M*_age_ = 75.3, *U* = 5773.00, *z* = −1.78, *p* = 0.007; *U* = 14068.00, *z* = −8.68, *p* < 0.001). Among the care-recipients with Alzheimer’s disease, caregivers predominantly took care of their partners (*p* < 0.001), in most cases a woman [χ^2^(1) = 18.907, *p* < 0.001]. Care-recipient degree of autonomy was also significantly lower than the other care-recipients (*M* = 10.7 vs. *M* = 19.6, *U* = 19953.00, *z* = −4.91, *p* < 0.001). Finally, caregivers of people with Parkinson’s and vascular dementia spent significantly fewer years providing care to their relatives compared to the other caregivers (*M*_years_ = 6.9 and *M*_years_ = 7.4 vs. *M*_years_ = 11.8, *U* = 4706.00, *z* = −3.18, *p* = 0.002; *U* = 6158.00, *z* = −2.70, *p* = 0.007). The rest of the sociodemographic and care situation variables did not have a significant relationship with any of the care-recipient diseases. No significant differences were found in self-esteem or social support of caregivers.

**Table 2:** Relationship between each care-recipient disease and caregiver emotional distress.

Disease of the care-recipient	Raw regression				Adjusted regression			
	*B*	*t*/Wald	*p*	Bayes factor	*B*	*t*/Wald	*p*	Bayes factor
**Blindness**								
Global emotional distress	2.496	1.044	0.297	0.062	3.052	1.258	0.209	<0.001
Somatic symptoms	0.891	1.208	0.228	0.074	1.113	1.488	0.137	<0.001
Anxiety-insomnia	0.095	0.094	0.925	0.036	0.343	0.336	0.737	<0.001
Social dysfunction	0.707	1.356	0.176	0.090	0.627	1.183	0.237	<0.001
Depression	0.803	1.228	0.220	0.076	0.969	1.463	0.144	<0.001
Probable mental disorder case	−0.721	0.413	0.521	0.152	−0.580	0.261	0.616	0.142
**Stroke**								
Global emotional distress	0.123	0.146	0.884	0.036	0.289	0.339	0.735	<0.001
Somatic symptoms	0.100	0.386	0.699	0.039	0.169	0.644	0.520	<0.001
Anxiety-insomnia	−0.247	−0.697	0.486	0.046	−0.170	−0.474	0.636	<0.001
Social dysfunction	−0.090	−0.491	0.624	0.041	−0.064	−0.342	0.733	<0.001
Depression	0.359	1.563	0.119	0.121	0.354	1.523	0.129	<0.001
Probable mental disorder case	0.231	0.502	0.479	0.042	0.302	0.836	0.361	0.049
**Hearth disease**								
Global emotional distress	−0.677	−0.469	0.639	0.040	−0.672	−0.465	0.642	<0.001
Somatic symptoms	−0.327	−0.734	0.463	0.047	−0.330	−0.738	0.461	<0.001
Anxiety-insomnia	0.096	0.159	0.874	0.036	0.112	0.184	0.854	<0.001
Social dysfunction	0.029	0.093	0.926	0.036	0.035	0.110	0.912	<0.001
Depression	−0.476	−1.205	0.229	0.074	−0.489	−1.237	0.217	0.001
Probable mental disorder case	−0.251	0.176	0.675	0.067	−0.250	0.173	0.677	0.067
**Chronic obstructive pulmonary disease**								
Global emotional distress	2.620	0.851	0.395	0.052	2.753	0.882	0.378	<0.001
Somatic symptoms	0.686	0.721	0.471	0.047	0.702	0.728	0.467	<0.001
Anxiety-insomnia	0.765	0.589	0.556	0.043	0.880	0.669	0.504	<0.001
Social dysfunction	0.436	0.648	0.517	0.044	0.480	0.704	0.482	<0.001
Depression	0.733	0.869	0.385	0.052	0.692	0.810	0.418	<0.001
Probable mental disorder case	1.374	1.251	0.263	0.260	1.425	1.315	0.252	0.260
**Kidney disease**								
Global emotional distress	2.496	1.044	0.297	0.062	2.681	1.115	0.265	<0.001
Somatic symptoms	0.487	0.659	0.510	0.045	0.530	0.714	0.476	<0.001
Anxiety-insomnia	0.297	0.294	0.769	0.038	0.376	0.371	0.711	<0.001
Social dysfunction	0.707	1.356	0.176	0.090	0.736	1.403	0.161	<0.001
Depression	1.005	1.538	0.125	0.117	1.039	1.580	0.115	0.001
Probable mental disorder case	0.269	0.086	0.769	0.100	0.291	0.099	0.753	0.086
**Arthritis**								
Global emotional distress	0.379	0.223	0.824	0.037	0.410	0.239	0.811	<0.001
Somatic symptoms	−0.018	−0.034	0.973	0.036	−0.007	−0.013	0.989	<0.001
Anxiety-insomnia	−0.313	−0.437	0.663	0.040	−0.363	−0.503	0.615	<0.001
Social dysfunction	0.102	0.274	0.784	0.037	0.078	0.210	0.834	<0.001
Depression	0.607	1.306	0.192	0.084	0.702	1.503	0.133	0.004
Probable mental disorder case	0.272	0.174	0.677	0.073	0.224	0.116	0.733	0.073
**Physical disability**								
Global emotional distress	−0.907	−0.606	0.545	0.043	−0.793	−0.521	0.602	<0.001
Somatic symptoms	0.069	0.149	0.882	0.036	0.048	0.103	0.918	<0.001
Anxiety-insomnia	−0.694	−1.103	0.271	0.066	−0.594	−0.929	0.354	<0.001
Social dysfunction	−0.135	−0.412	0.681	0.039	−0.067	−0.203	0.839	<0.001
Depression	−0.147	−0.359	0.719	0.038	−0.180	−0.432	0.666	<0.001
Probable mental disorder case	−0.143	0.055	0.815	0.064	−0.048	0.006	0.938	0.064
**Cancer**								
Global emotional distress	0.274	0.125	0.900	0.036	0.106	0.048	0.961	0.003
Somatic symptoms	1.366	2.030	0.053	0.279	1.327	1.963	0.051	0.019
Anxiety-insomnia	−0.749	−0.814	0.416	0.050	−0.801	−0.867	0.387	0.003
Social dysfunction	−0.068	−0.142	0.887	0.036	−0.079	−0.164	0.869	0.002
Depression	−0.275	−0.459	0.646	0.040	−0.340	−0.566	0.572	0.004
Probable mental disorder case	0.681	0.686	0.408	0.119	0.672	2.015	0.055	0.119
**Spina bifida**								
Global emotional distress	−3.607	−1.654	0.099	0.140	−3.846	−1.709	0.088	<0.001
Somatic symptoms	−0.828	−1.227	0.220	0.076	−0.755	−1.089	0.277	0.001
Anxiety-insomnia	−1.930	0.917	0.056	0.325	−1.922	−2.033	0.052	0.001
Social dysfunction	−0.574	−1.204	0.229	0.074	−0.564	−1.147	0.252	<0.001
Depression	−0.275	−0.459	0.646	0.040	−0.605	−0.982	0.326	<0.001
Probable mental disorder case	−0.947	0.742	0.389	0.183	−0.820	0.532	0.466	0.165
**Down syndrome**								
Global emotional distress	−0.412	−0.424	0.672	0.039	−0.211	−0.188	0.851	<0.001
Somatic symptoms	−0.507	−1.691	0.091	0.149	−0.439	−1.272	0.204	<0.001
Anxiety-insomnia	−0.201	−0.490	0.625	0.040	0.108	0.230	0.818	<0.001
Social dysfunction	0.107	.0503	0.615	0.041	0.204	0.832	0.406	<0.001
Depression	0.189	0.708	0.479	0.046	−0.084	−0.273	0.785	<0.001
Probable mental disorder case	−0.454	1.170	0.280	0.079	−0.293	0.361	0.548	0.060
**Angelman syndrome**								
Global emotional distress	−1.582	−0.724	0.470	0.047	−1.938	−0.849	0.396	<0.001
Somatic symptoms	−0.490	−0.726	0.468	0.047	−0.472	0.671	0.503	0.001
Anxiety-insomnia	−0.243	−0.263	0.792	0.037	−0.192	−0.199	0.842	<0.001
Social dysfunction	−0.405	−0.850	0.396	0.051	−0.377	−0.757	0.450	<0.001
Depression	−0.444	−0.741	0.459	0.047	−0.898	−1.444	0.149	0.001
Probable mental disorder case	−0.947	0.742	0.389	0.184	−0.289	0.270	0.603	0.182
**Cat’s cry syndrome**								
Global emotional distress	5.674	2.613	0.059	0.758	5.718	2.530	0.052	0.001
Somatic symptoms	1.366	2.030	0.053	0.279	1.431	2.054	0.051	0.003
Anxiety-insomnia	1.445	1.573	0.116	0.123	1.528	1.600	0.110	<0.001
Social dysfunction	1.919	3.238	0.001	1.256	1.654	2.683	0.008	1.001
Depression	0.945	1.987	0.057	0.890	1.106	2.237	0.056	0.001
Probable mental disorder case	0.681	0.686	0.408	0.119	0.796	0.853	0.356	0.138
**Schizophrenia**								
Global emotional distress	−0.708	−0.583	0.560	0.043	−0.038	−0.607	0.544	0.003
Somatic symptoms	−0.123	−0.327	0.744	0.038	−0.131	−0.350	0.727	0.003
Anxiety-insomnia	−0.215	−0.420	0.674	0.039	−0.223	−0.436	0.663	0.003
Social dysfunction	−0.365	−1.379	0.169	0.093	−0.367	−1.385	0.167	0.005
Depression	−0.005	−0.015	0.988	0.036	−0.016	−0.048	0.962	0.004
Probable mental disorder case	−0.443	0.712	0.399	0.078	−0.447	3.358	0.067	0.078
**Bipolar disorder**								
Global emotional distress	−0.063	−0.020	0.984	0.036	−0.120	−0.039	0.969	0.003
Somatic symptoms	0.016	0.016	0.987	0.036	−0.001	−0.001	0.999	0.003
Anxiety-insomnia	−0.241	−0.186	0.853	0.037	−0.257	−0.198	0.843	0.002
Social dysfunction	−0.906	−1.349	0.178	0.089	−0.910	−1.353	0.177	0.005
Depression	1.068	1.267	0.206	0.080	1.048	1.243	0.215	0.008
Probable mental disorder case	−0.672	2.275	0.051	0.133	−0.027	0.001	0.983	0.133
**Autism**								
Global emotional distress	−2.189	−1.081	0.280	0.064	−2.423	−1.174	0.241	<0.001
Somatic symptoms	−0.322	−0.515	0.607	0.041	−0.350-	0.552	0.581	<0.001
Anxiety-insomnia	−1.064	−1.249	0.212	0.078	−1.051	−1.212	0.226	<0.001
Social dysfunction	−0.189	−0.426	0.670	0.039	−0.192	−0.426	0.670	<0.001
Depression	−0.614	−1.107	0.269	0.066	−0.829	−1.474	0.141	<0.001
Probable mental disorder case	−14.916	0.001	0.978	7.933	−20.542	0.001	0.999	7.405
**Rett syndrome**								
Global emotional distress	−0.398	−0.129	0.897	0.036	0.255	0.081	0.935	<0.001
Somatic symptoms	−0.655	−0.688	0.492	0.045	−0.400	−0.413	0.680	<0.001
Anxiety-insomnia	1.100	0.848	0.397	0.051	1.707	1.298	0.195	0.002
Social dysfunction	−0.235	−0.349	0.727	0.038	−0.164	−0.238	0.812	<0.001
Depression	−0.609	−0.721	0.471	0.047	−0.888	−1.034	0.302	<0.001
Probable mental disorder case	−0.021	0.001	0.986	0.133	0.412	0.107	0.744	0.145
**West syndrome**								
Global emotional distress	−1.141	−0.477	0.634	0.040	−0.641	−0.264	0.792	<0.001
Somatic symptoms	−1.129	−1.531	0.126	0.115	−0.929	−1.241	0.215	<0.001
Anxiety-insomnia	−1.118	−1.111	0.267	0.066	−0.746	−0.732	0.464	0.001
Social dysfunction	0.505	0.968	0.334	0.057	0.585	1.101	0.272	<0.001
Depression	0.601	0.918	0.359	0.055	0.449	0.675	0.500	<0.001
Probable mental disorder case	−0.721	0.413	0.521	0.152	−0.510	0.861	0.353	0.144
**Cerebral palsy**								
Global emotional distress	−0.814	−1.034	0.301	0.061	−0.805	−1.024	0.307	0.005
Somatic symptoms	−0.180	−0.738	0.461	0.047	−0.177	−0.728	0.467	0.004
Anxiety-insomnia	−0.224	−0.675	0.500	0.045	−0.221	−0.667	0.505	0.003
Social dysfunction	−0.129	−0.753	0.452	0.048	−0.129	−0.749	0.454	0.003
Depression	−0.281	−1.306	0.192	0.084	−0.278	−1.292	0.197	0.009
Probable mental disorder case	−0.444	1.737	0.187	0.085	−0.471	1.918	0.166	0.085
**Multiple sclerosis**								
Global emotional distress	−1.751	−0.801	0.423	0.049	−1.734	−0.793	0.428	0.004
Somatic symptoms	−0.659	−0.977	0.329	0.058	−0.654	−0.969	0.333	0.004
Anxiety-insomnia	−0.074	−0.080	0.936	0.036	−0.069	−0.075	0.940	0.002
Social dysfunction	−0.405	−0.850	0.396	0.051	−0.404	−0.847	0.398	0.003
Depression	−0.612	−1.024	0.306	0.061	−0.606	−1.014	0.311	0.006
Probable mental disorder case	−0.022	0.001	0.980	0.091	−0.020	0.001	0.982	0.091
**Parkinson**								
Global emotional distress	−0.255	0.258	0.797	0.037	0.069	0.069	0.945	<0.001
Somatic symptoms	−0.408	−1.339	0.181	0.088	−0.505	−1.640	0.102	<0.001
Anxiety-insomnia	0.307	0.737	0.461	0.047	0.167	0.399	0.690	0.001
Social dysfunction	0.141	0.654	0.513	0.044	0.122	0.556	0.579	<0.001
Depression	0.215	0.796	0.427	0.049	0.285	1.041	0.298	<0.001
Probable mental disorder case	0.371	0.967	0.325	0.061	0.260	0.461	0.497	0.050
**Epilepsy**								
Global emotional distress	4.207	2.724	0.057	0.732	3.224	1.735	0.053	0.116
Somatic symptoms	0.699	1.458	0.145	0.104	0.704	1.468	0.143	0.008
Anxiety-insomnia	1.036	1.585	0.114	0.125	1.040	1.591	0.112	0.008
Social dysfunction	1.515	3.604	0.001	1.945	0.958	2.840	0.005	1.113
Depression	0.957	2.840	0.055	0.736	1.522	2.620	0.051	0.112
Probable mental disorder case	0.344	0.336	0.562	0.071	−0.620	3.765	0.052	0.071
**Alzheimer**								
Global emotional distress	0.100	0.197	0.844	0.037	0.163	0.291	0.771	<0.001
Somatic symptoms	0.157	1.002	0.317	0.059	0.173	1.005	0.315	<0.001
Anxiety-insomnia	0.305	1.431	0.153	0.100	0.306	1.308	0.192	<0.001
Social dysfunction	−0.040	−0.364	0.716	0.038	−0.067	−0.546	0.585	<0.001
Depression	−0.322	−2.330	0.120	0.092	−0.250	−1.636	0.102	<0.001
Probable mental disorder case	0.276	1.919	0.166	0.052	−0.439	0.559	0.455	0.050
**Vascular dementia**								
Global emotional distress	0.088	0.097	0.923	0.036	−0.013	−0.015	0.988	<0.001
Somatic symptoms	0.241	0.858	0.391	0.052	0.193	0.680	0.497	<0.001
Anxiety-insomnia	0.043	0.112	0.911	0.036	−0.060	−0.155	0.877	0.001
Social dysfunction	−0.009	−0.045	0.964	0.036	−0.024	−0.121	0.904	<0.001
Depression	−0.187	−0.749	0.454	0.048	−0.122	−0.486	0.627	<0.001
Probable mental disorder case	−0.067	0.034	0.854	0.037	−0.159	0.185	0.667	0.041

*Adjusted regression was controlled for the relationship with the care-receipt and for the sociodemographic and care-related variables that were significant for each disease in previous analyses.*

The relationship between each of the diseases and the global emotional distress, its four subscales, and the percentage of probable case of mental disorder were examined (Table 2). A significant relationship was found only between caring for people with cat’s cry syndrome and epilepsy and caregiver social dysfunction (*B* = 1.919, *p* = 0.001; *B* = 1.515, *p* = 0.001). There was no relation in global emotional distress, somatic symptoms, anxiety and insomnia, social dysfunction, depression and probable cases of mental disorder with the other care-recipient diagnosis. After adjusting for the relationship and the variables that were significant in the previous analysis, the results remained the same.

The Bayes factor analysis corroborated the results above, except for caregivers for people with autism. As Table 2 shows, the Bayes factors were 1.256 in social dysfunction for caregivers of people with cat’s cry syndrome and 1.945 for caregivers of people with epilepsy [which according to Jeffreys (1961) guidelines provide anecdotal evidence for the alternative hypothesis]. A Bayes factor of 7.933 was found for probable mental health case for caregivers of care-recipients with autism, indicating substantial evidence for the alternative hypothesis. The rest of the diseases yielded Bayes factors between 0.036 and 0.758, which provides anecdotal to strong evidence for the null hypothesis. After adjusting for relationship and the previously significant variables, the Bayes factors for cat’s cry syndrome, epilepsy and autism were slightly reduced to 1.001, 1.113, and 7.405, respectively, providing anecdotal to substantial evidence for the alternative hypothesis. For the rest of the diseases, Bayes factors ranged from <0.001 to 0.260, denoting substantial to extreme evidence for the null hypothesis.

### Relationship of the Process Stress Variables in the Appearance of the Emotional Distress of the Caregiver

The adjustment indexes of the fit between the theoretical model by Pearlin et al. (1990) and the observed data were as follows: χ^2^(103) = 283.30, *p* < 0.001; χ^2^/df = 2.75; GFI = 0.94; CFI = 0.82; RMSEA = 0.059; SRMR = 0.061, indicating that the model had an acceptable fit to the data.

**FIGURE 2:**
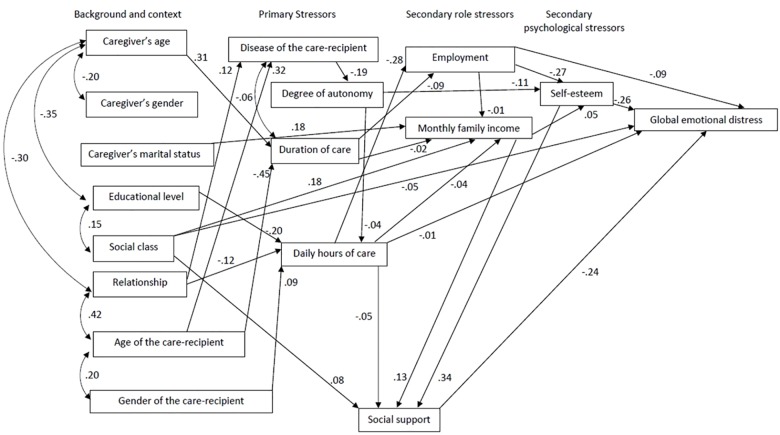
Relationships of the process stress variables on caregiver emotional state.

As Figure 2 shows, there are significant relationships between certain sociodemographic variables and primary stressors, between some primary and secondary stressors, and between the secondary stressor of self-esteem and global emotional distress. Specifically, we see that caregivers’ age was positively related to duration of care (β = 0.31, *p* < 0.001), educational level was negatively related to daily hours of care (β = −0.20, *p* < 0.001), and age of care-recipient was associated with care-recipient disease (β = 0.32, *p* < 0.001), with older recipients being more likely to suffer from dementia. The care-recipient’s disease influenced their level of autonomy, with care-recipients with dementia demonstrating less autonomy (β = −0.19, *p* < 0.001). In addition, daily hours of care were negatively related to having a job (β = −0.28, *p* < 0.001), and the care-recipient autonomy was negatively related to caregiver’s self-esteem (β = −0.11, *p* < 0.001). Having a job was negatively related to self-esteem (β = −0.27, *p* < 0.001) and monthly family income was positively related to social support (β = 0.13, *p* < 0.001). Finally, caregiver’s self-esteem was positively related to social support (β = 0.34, *p* < 0.001) and negatively related to the global emotional distress (β = −0.26, *p* < 0.001).

In addition, we found that the weight of the regression for daily hours of care (B1 = −0.081) explains 1% of the variance. When including social support, this weight decreased (B1′ = −0.164), which indicates that it is acting as a mediating variable for the relationship between daily hours of care and global emotional distress. The Sobel test corroborates this, showing a significant effect from this mediation [*Z*(Sobel) = −2.13, *p* = 0.032]. The quotient B1 - B1′/B1′ = 0.50 indicates that 50% of the relationship between daily hours of care and global emotional distress is explained by social support. Social support also mediates the relationship between social class and global emotional distress because the weight of the regression decreases when including the mediating variable (B1 = −1.13, B1′ = −1.61). *Z* = −2.56; *p* = 0.010. The B1 - B1′/B1′ = 0.29 ratio indicates that 29% of the relationship between social class and global emotional distress is explained by social support. Social support also mediates the relationship between monthly family income and global emotional distress; that is, the weight of the regression decreases when including the mediating variable (B1 = −0.381, B1′ = −1.66). *Z* = −3.66; *p* < 0.001. The B1 - B1′/B1′ = 0.76 ratio indicates that 76% of the relationship between monthly family income and global emotional distress is explained by social support. Finally, social support also mediates the relationship between self-esteem and global emotional distress. The regression weight decreases when social support is included (B1 = −0.440, B1′ = −1.20). *Z* = −8.31; *p* = 0.010. The B1 - B1′/B1′ = 0.63 ratio indicates that 63% of the self-esteem and global emotional distress is explained by social support.

## Discussion

The objectives of this study were (a) to determine the caregivers’ emotional state, (b) to analyze the association between each care-recipient disease and the emotional state of the caregiver, and (c) to analyze the relationship of the process stress variables in the appearance of the emotional distress of the caregiver based on the theoretical model by Pearlin et al. (1990). The results indicated that the caregivers showed moderate emotional distress. This emotional impact was similar in caregivers of care-recipients with different diseases, except when they took care for a care-recipient with cat’s cry syndrome or epilepsy (which was related to suffering from social dysfunction), and a care-recipient with autism (related to having a probable mental health case). Social support mediated the relationship between social class, daily hours of care, self-esteem and global emotional distress.

### Emotional State of the Caregivers

Overall, the caregivers had moderate emotional distress (somatic, anxiety and insomnia symptoms had the highest scores), and 33.8% presented with a probable mental disorder. This finding is consistent with previous research (Vázquez et al., 2018), where psychopathological symptoms were common among caregivers, and anxiety symptoms were one of the most frequently observed.

### Association Between Care-Recipient Diseases and Caregiver Emotional State

In the current study, we found that caregivers of stroke care-recipients were most likely to be women and that caregivers of care-recipients with blindness were older compared to caregivers caring for people suffering from other diseases. Caregivers of people with Angelman syndrome and cat’s cry syndrome were younger. Care-recipients having spina bifida, Down syndrome, Angelman syndrome, and cat’s cry syndrome, autism, Rett and West syndromes were younger, and most likely to be the children of the caregivers. Care-recipients with Down syndrome were most likely to be men. The caregivers caring for a care-recipient having Down syndrome or Rett syndrome provided care for the longest duration, typically since birth. Care-recipients who suffered from Parkinson’s and Alzheimer’s were significantly older, and in Alzheimer’s disease most likely to be the wife of the caregiver, and with a high level of dependency. Lastly, Parkinson’s and vascular dementia caregivers took care of their relative for a shorter period. These findings are consistent with the age of onset, gender prevalence, life expectancy and clinical profiles of the different diseases (e.g., Yang et al., 2002; Statistic National Institute, 2008; Podcasy and Epperson, 2016) and indicates there are likely differences in care scenarios depending on the disease of the care-recipient.

However, with and without adjusting for the relationship and the sociodemographic and care-related variables, taking care for a care-recipient with cat’s cry syndrome and epilepsy were related to the caregivers’ social dysfunction. These findings could be explained by the striking characteristic symptoms of the particular diseases. For example, the cry of children with cat’s cry syndrome or the symptoms of epileptic seizures could limit caregivers’ social relationships, although this result should be taken with caution since the data only provide anecdotal evidence according to the Bayes factor. In addition, a Bayes factor analysis found that the evidence substantially favored the alternative hypothesis about caring for a care-recipient with autism was related to having a probable mental health case. This finding is consistent with previous studies (Bromley et al., 2004; Herrema et al., 2017) and could be explained due to the higher levels of challenging behavior and aggressiveness of autistic care-recipients. Regarding the other care-recipient’s diseases, there were no differences in the global emotional distress, no in the symptoms of somatization, anxiety-insomnia, social dysfunction and depression, nor in the percentage of probable cases of mental disorder, which reach extreme evidence according to Bayes factor. These findings are consistent with those of Crespo et al. (2005), where there was no evidence that caregivers of dementia patients had poorer emotional state than those caring for a care-recipient without cognitive impairment. However, our findings differ from those obtained by Clipp and George (1993) and Ory et al. (1999), where dementia caregivers presented more adverse psychological effects than caregivers of people with other diseases, and those obtained by Papastavrou et al. (2012) and Loi et al. (2015) where cancer and physical disease caregivers showed more psychological distress. However, these comparations should be taken with caution due to differences on the sample characteristics. In the previous studies caregivers belonged to convenience samples (Clipp and George, 1993; Papastavrou et al., 2012; Loi et al., 2015) and focused on the comparison of specific diseases such as dementia vs. cancer (Clipp and George, 1993), dementia vs. non-dementia (Ory et al., 1999), dementia vs. older age without cognitive impairment (Crespo et al., 2005) or dementia vs. schizophrenia and cancer (Papastavrou et al., 2012). They also selected specific caregivers related to the care-recipient such as spouses (Clipp and George, 1993), caregivers caring for a care-recipient who was at least 50 (Ory et al., 1999) or 60 (Crespo et al., 2005; Loi et al., 2015) years of age.

### Relationship of the Process Stress Variables in the Caregiver’s Emotional Distress

When considering the background and context variables, a significant positive relationship was found between caregiver’s age and duration of care and between care-recipient’s age and the disease suffered; a negative relation between educational level and the daily hours of care. A possible explanation for this is that the age of the caregiver increases as the years taking care of the care-recipients increases. Furthermore, the older the care-recipient, the more likely they were to have dementia, likely because the onset of this disease typically affects those older in age. In addition, a possible explanation for the negative relationship between educational level and hours of care is that relatives with lower education are likely to be those who are housewives or jobs with less than ideal conditions, making them most likely to be dedicated to providing many hours of care to the care-recipients.

When considering the primary stressors, those with dementia were more likely to have a lower level of autonomy, and the care-recipients’ autonomy was negatively related to caregivers’ self-esteem. In addition, hours of daily care were negatively related to having a job. The relationship between dementia and lower level of autonomy is consistent with the literature, because dementia has been found to produce a leading cause of disability-adjusted life-years (GBD 2015 Neurological Disorders Collaborator Group, 2017). A possible explanation for the findings regarding caregivers’ self-esteem is that care-recipients with greater dependence would limit the personal development of the caregiver by restricting their time away, neglecting their personal care or giving up their job. Furthermore, the number of daily hours dedicated to care also limits their labor opportunities. As a result, the caregivers may not feel completely self-realized, decreasing their self-esteem.

On the other hand, when considering secondary role stressors, having a job was found to be negatively related to caregiver’s self-esteem. This is likely caused by an emotional conflict experienced by the caregivers; that is, after being out of the labor market for years they may feel insecure at a job, and when they go to work, they feel guilty for not attending to the care-recipient. Furthermore, monthly family income was positively related to social support. A possible explanation for this is that caregivers with greater income can access some social activities that require financial stability (e.g., making certain social activities such as having dinner in a restaurant, going to the cinema or drinking coffee in a coffee shop, or even paying someone to take care of their relative while they go out with friends).

The assessed secondary psychological stressor, self-esteem, was positively related to social support and negatively to global emotional distress. Having a good self-esteem is necessary for good quality of social relationships, because insecurities are often characterized by self-deprecating comments and the insecurities can make social relationships difficult. In addition, the finding that self-esteem is related to emotional distress is consistent with previous research which found that self-esteem is related to anxiety and depression (Garaigordobil et al., 2008).

Furthermore, social support mediated the relationship between social class, daily hours dedicated to care, monthly family income, self-esteem, and global emotional distress, explaining 29%, 50%, 76%, and 63% of the effect of these variables on emotional distress, respectively. We hypothesized that social support can help those caregivers from a lower class overcome difficulties derived from dedicate large hours to care, have low family income and low self-esteem by providing instrumental, economic and emotional assistance to overcome these difficulties, thus providing them with resources and compensating their deficits. Additionally, this finding is consistent with previous work that has identified that social support can act as a buffer against mental health problems (Olstad et al., 2001).

These findings are consistent with the premise that caregiver’s emotional distress arises as a result of the interaction between not only primary stressors but also the appraisal style and resources in managing stressors according to the cognitive stress theory (Lazarus and Folkman, 1984) and the process stress model (Pearlin et al., 1990). This may explain why some caregivers become depressed whereas others are less depressed under similar caregiving situations.

### Implications

This work has important implications for research and social and health policies. For the caregivers of care-recipients with a number of diseases, it is suggested that research and psychological interventions to caregivers should focus on the caregiver and their psychological resources and not only on the disease of the person cared, in accordance with the recommendations from Zarit and Femia (2008). In fact, not all the caregivers experience negative consequences of caring; some caregivers report positive aspects of caring, experiencing life satisfaction and wellbeing (Lawton et al., 1991; Cohen et al., 2002). The present findings demonstrate an exploratory but empirically validated model for the appearance of emotional distress in caregivers. Future research can further assess the causal variables (i.e., direct and indirect effects, moderators and mediators) on caregiver’s emotional distress. In addition, these findings suggest that psychological intervention to caregivers can be improved by performing evaluations that are not limited to the care situation, by including a complete set of variables which must include background and context (i.e., the caregiver’s age, educational level, social class and age of the care-recipient), primary stressors (i.e., disease of the care-recipient, degree of autonomy and daily hours of care), secondary role stressors (i.e., monthly family income), secondary psychological stressors (i.e., self-esteem) and psychological resources (social support). They also can be improved by selecting caregivers based on their symptoms, needs, self-esteem and resources such social support instead of directing interventions to all caregivers of people with a certain disease (e.g., dementia caregivers) assuming that all of them experience a negative emotional state, and designing tailored interventions where caregivers are trained in psychological skills that act as protective factors (social support). Following these guidelines may have contributed to larger effect sizes found in the interventions of Otero et al. (2015) and Vázquez et al. (2016). Specifically in caregivers of care-recipients suffering from cat’s cry syndrome, epilepsy and autism, psychological interventions may need to include a higher number of sessions or additional components, such as assertiveness training and/or psychoeducational interventions to improve caregivers’ social functioning in caregivers of care-recipients with cat’s cry syndrome and epilepsy, or respite care in caregivers of care-recipients with autism to avoid a mental disorder onset.

### Limitations

Some limitations in this study must be considered. These results are cross-sectional, so it is not possible to make causal inferences and it does not consider the dynamic nature of the care trajectory in which caregivers mental health experience fluctuations over time. These issues could be addressed in longitudinal studies. Several categories of diseases included few participants, and thus the results should be considered with caution. Replication of these findings in future studies with large sample sizes is recommended. Although the most important variables of the theoretical model of Pearlin et al. (1990) are included in this study, it was not possible to include all of them exhaustively. Future research should also consider other variables not evaluated here, such as coping, as a potential mediating variable.

Despite these limitations, this is the first study to include care-recipients with all types of diseases indicated in the ICD-10 classification in a randomly selected sample of an adequate size, in which reliable and valid outcome measures were used.

## Ethics Statement

This study was carried out in accordance with the recommendations of World Health Organization guidelines and the bioethics committee of the University of Santiago de Compostela (Spain). The protocol was approved by the bioethics committee of the University of Santiago de Compostela. All participants gave written informed consent in accordance with the Declaration of Helsinki.

## Author Contributions

PO and FV contributed to the conception and design of the study and wrote the first draft of the manuscript. PO, FV, and MF performed the statistical analysis. VB, ÁT, and OD reviewed the text critically. PO, VB, and OD participated in the investigation. All authors contributed to manuscript revision, read and approved the submitted version.

## Conflict of Interest Statement

The authors declare that the research was conducted in the absence of any commercial or financial relationships that could be construed as a potential conflict of interest.
